# Supplementary material for: A small number of workers with specific personality traits perform tool use in ants
Source: eLife. 2020 Dec 9;9:e61298. doi: 10.7554/eLife.61298 (PMC7725502; doi:10.7554/eLife.61298)

A small number of workers with specific personality traits perform tool use in ants

István Maák, Garyk Roelandt, Patrizia d’Ettorre

2020 09 24

**Experiment 1 - Tool use process in whole colonies**

library(lme4)

library(MuMIn)

library(lmerTest)

library(rsq)

library(effsize)

library(readxl)

knitr::opts_chunk$set(cache =TRUE)

*Model testing the effect of the number of workers in the foraging arena on the latency of the first tool dropped to the bait:*

Exp1=read_excel("C:/Users/NITRO/Desktop/RMarkDown/Data_Experiment1.xlsx",sheet=1)

colID=with(Exp1,as.factor(ColID))
m1=lmer(log(Firsttoolbait)~Workinvolv+Workinarena+(1|colID),data=Exp1,na.action="na.fail")
summary(m1)

## Linear mixed model fit by REML. t-tests use Satterthwaite's method [
## lmerModLmerTest]
## Formula: log(Firsttoolbait) ~ Workinvolv + Workinarena + (1 | colID)
## Data: Exp1
##
## REML criterion at convergence: 47.6
##
## Scaled residuals:
## Min 1Q Median 3Q Max
## -1.49723 -0.35171 0.00063 0.30825 1.79285
##
## Random effects:
## Groups Name Variance Std.Dev.
## colID (Intercept) 0.4719 0.6869
## Residual 0.8187 0.9048
## Number of obs: 15, groups: colID, 3
##
## Fixed effects:
## Estimate Std. Error df t value Pr(>|t|)
## (Intercept) 2.21957 0.91227 7.36419 2.433 0.0435 *
## Workinvolv 0.37753 0.28098 11.83986 1.344 0.2043
## Workinarena -0.03402 0.01530 11.71458 -2.223 0.0467 *
## ---
## Signif. codes: 0 '***' 0.001 '**' 0.01 '*' 0.05 '.' 0.1 ' ' 1
##
## Correlation of Fixed Effects:
## (Intr) Wrknvl
## Workinvolv -0.771
## Workinarena -0.488 0.135

Running model diagnostics plot:

plot(m1)


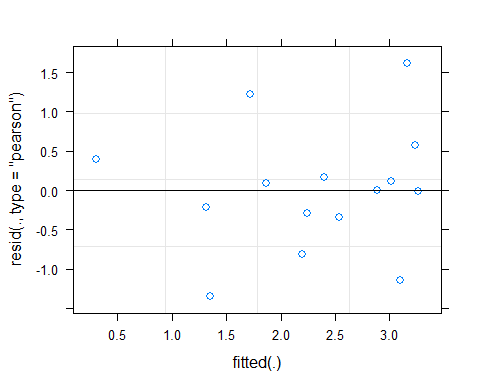


*Model testing the effect of latency to drop first tool on total time of tool transport to the bait:*

m2=lmer(log(Tottime)~Workinvolv+Firsttoolbait+(1|colID),data=Exp1,na.action="na.fail")
summary(m2)

## Linear mixed model fit by REML. t-tests use Satterthwaite's method [
## lmerModLmerTest]
## Formula: log(Tottime) ~ Workinvolv + Firsttoolbait + (1 | colID)
## Data: Exp1
##
## REML criterion at convergence: 31.5
##
## Scaled residuals:
## Min 1Q Median 3Q Max
## -1.4548 -0.5704 0.1263 0.5185 1.3197
##
## Random effects:
## Groups Name Variance Std.Dev.
## colID (Intercept) 0.1214 0.3485
## Residual 0.1942 0.4407
## Number of obs: 15, groups: colID, 3
##
## Fixed effects:
## Estimate Std. Error df t value Pr(>|t|)
## (Intercept) 2.82148 0.39251 7.39105 7.188 0.000138 ***
## Workinvolv -0.06561 0.14121 11.97631 -0.465 0.650546
## Firsttoolbait 0.01013 0.00448 11.86073 2.261 0.043380 *
## ---
## Signif. codes: 0 '***' 0.001 '**' 0.01 '*' 0.05 '.' 0.1 ' ' 1
##
## Correlation of Fixed Effects:
## (Intr) Wrknvl
## Workinvolv -0.777
## Firsttoolbt -0.005 -0.270

Running model diagnostics plot:

plot(m2)


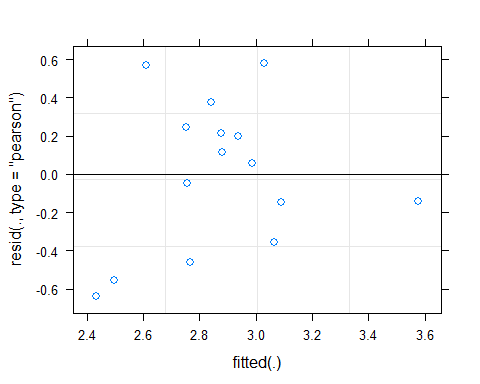


*Model testing the effect of the dynamics of tool transport to the bait (latency to drop the first*
*tool into bait, total time of tool transport, number of workers involved in tool use) on the*
*latency of transport the first tool to the nest:*

tri=with(Exp1,as.factor(Trial))
m3=lmer(log(Firsttoolnest+1)~Firsttoolbait+Tottime+Workinvolv+(1|colID)+(1|tri),data=Exp1,na.action="na.fail")

## boundary (singular) fit: see ?isSingular

summary(m3)

## Linear mixed model fit by REML. t-tests use Satterthwaite's method [
## lmerModLmerTest]
## Formula: log(Firsttoolnest + 1) ~ Firsttoolbait + Tottime + Workinvolv +
## (1 | colID) + (1 | tri)
## Data: Exp1
##
## REML criterion at convergence: 73.4
##
## Scaled residuals:
## Min 1Q Median 3Q Max
## -1.4638 -0.7556 0.2964 0.6778 1.0033
##
## Random effects:
## Groups Name Variance Std.Dev.
## tri (Intercept) 6.811e-08 0.000261
## colID (Intercept) 0.000e+00 0.000000
## Residual 6.570e+00 2.563252
## Number of obs: 15, groups: tri, 5; colID, 3
##
## Fixed effects:
## Estimate Std. Error df t value Pr(>|t|)
## (Intercept) 5.98429 2.44888 11.00000 2.444 0.0326 *
## Firsttoolbait 0.02507 0.02518 11.00000 0.996 0.3408
## Tottime -0.12121 0.09014 11.00000 -1.345 0.2058
## Workinvolv -0.31206 0.70421 11.00000 -0.443 0.6663
## ---
## Signif. codes: 0 '***' 0.001 '**' 0.01 '*' 0.05 '.' 0.1 ' ' 1
##
## Correlation of Fixed Effects:
## (Intr) Frsttl Tottim
## Firsttoolbt 0.143
## Tottime -0.676 -0.417
## Workinvolv -0.697 -0.074 0.057
## convergence code: 0
## boundary (singular) fit: see ?isSingular

Running model diagnostics plot:

plot(m3)


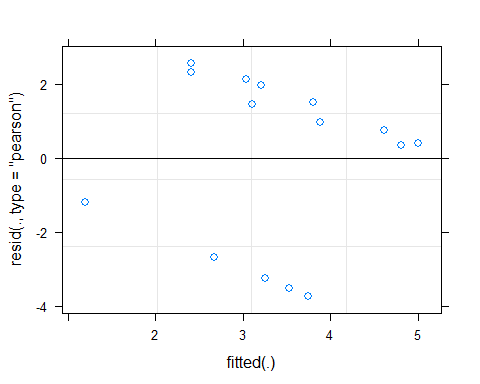


*Paint marked tool users. Correlation between the latency to obtain information about the food*
*and tools and the latency to drop the first tool on bait:*

Lat_corr=read_excel("C:/Users/NITRO/Desktop/RMarkDown/Data_Experiment1.xlsx",sheet=2)

with(Lat_corr,cor.test(Latcontacttoolfood,Latfirsttoolonbait,method="spearman"))

## Warning in cor.test.default(Latcontacttoolfood, Latfirsttoolonbait, method =
## "spearman"): Cannot compute exact p-value with ties

##
## Spearman's rank correlation rho
##
## data: Latcontacttoolfood and Latfirsttoolonbait
## S = 975, p-value = 0.9805
## alternative hypothesis: true rho is not equal to 0
## sample estimates:
## rho
## -0.006195148

**Experiment 2 - Is there specialization in tool use?**

library(rptR)

*The repeatability of the occurence of tool use:*

Rep_Tooluse=read_excel("C:/Users/NITRO/Desktop/RMarkDown/Data_Experiment2.xlsx",sheet=1)

m4=rpt(Tool.users~(1|ID.ant)+(1|Colony),
 grname="ID.ant", nboot = 0, npermut = 1000, datatype="Binary", data=Rep_Tooluse)

## boundary (singular) fit: see ?isSingular

## Permutation Progress for ID.ant:

## boundary (singular) fit: see ?isSingular

summary(m4)

##
## Repeatability estimation using glmer method
##
## Call = rpt(formula = Tool.users ~ (1 | ID.ant) + (1 | Colony), grname = "ID.ant", data = Rep_Tooluse, datatype = "Binary", nboot = 0, npermut = 1000)
##
## Data: 1880 observations
## ----------------------------------------
##
## ID.ant (480 groups)
##
## Repeatability estimation overview:
## R SE 2.5% 97.5% P_permut
## Org 0.212 NA NA NA 0.001
## Link 0.218 NA NA NA 0.001
##
##
## Bootstrapping:
## N Mean Median 2.5% 97.5%
## Org 1 NA NA NA NA
## Link 1 NA NA NA NA
##
## Permutation test:
## N Mean Median 2.5% 97.5% P_permut
## Org 1000 0.0155 4.04e-11 0 0.0605 0.001
## Link 1000 0.0155 4.04e-11 0 0.0607 0.001
##
## Likelihood ratio test:
## logLik full model = -459.0107
## logLik red. model = -488.0886
## D = 58.2, df = 1, P = 1.21e-14
##
## ----------------------------------------

*Model on the time required for the transport of one tool during consecutive transports by the same worker within a trail:*

Rep_withinTrial=read_excel("C:/Users/NITRO/Desktop/RMarkDown/Data_Experiment2.xlsx",sheet=2)

rep=with(Rep_withinTrial,as.factor(Rep))
colID=with(Rep_withinTrial,as.factor(ColID))
m5=lmer(log(TimeOneTool)~rep+(1|colID),data=Rep_withinTrial)
summary(m5)

## Linear mixed model fit by REML. t-tests use Satterthwaite's method [
## lmerModLmerTest]
## Formula: log(TimeOneTool) ~ rep + (1 | colID)
## Data: Rep_withinTrial
##
## REML criterion at convergence: 131.7
##
## Scaled residuals:
## Min 1Q Median 3Q Max
## -2.4555 -0.4828 -0.1751 0.6765 2.6901
##
## Random effects:
## Groups Name Variance Std.Dev.
## colID (Intercept) 0.2350 0.4848
## Residual 0.5699 0.7549
## Number of obs: 54, groups: colID, 6
##
## Fixed effects:
## Estimate Std. Error df t value Pr(>|t|)
## (Intercept) 4.3517 0.2731 8.1630 15.934 1.95e-07 ***
## rep2 -0.5652 0.2516 45.5085 -2.246 0.029607 *
## rep3 -0.9438 0.2516 45.5085 -3.750 0.000497 ***
## ---
## Signif. codes: 0 '***' 0.001 '**' 0.01 '*' 0.05 '.' 0.1 ' ' 1
##
## Correlation of Fixed Effects:
## (Intr) rep2
## rep2 -0.461
## rep3 -0.461 0.500

Running model diagnostics plot:

plot(m5)


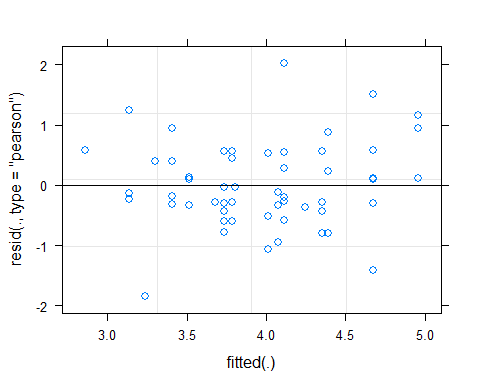


Calculating the Cohen.d to assess the effect size for the different factor levels:

rep1=subset(Rep_withinTrial,Rep=="1")
rep2=subset(Rep_withinTrial,Rep=="2")
rep3=subset(Rep_withinTrial,Rep=="3")

cohen.d(rep1$TimeOneTool,rep2$TimeOneTool,conf.level=0.95)

##
## Cohen's d
##
## d estimate: 0.4499891 (small)
## 95 percent confidence interval:
## lower upper
## -0.2359453 1.1359235

cohen.d(rep1$TimeOneTool,rep3$TimeOneTool,conf.level=0.95)

##
## Cohen's d
##
## d estimate: 0.9366506 (large)
## 95 percent confidence interval:
## lower upper
## 0.2230577 1.6502435

*Model to compare the average time needed to transport one tool by the same worker between the trials:*

Rep_betweenTrials=read_excel("C:/Users/NITRO/Desktop/RMarkDown/Data_Experiment2.xlsx",sheet=3)

rep=with(Rep_betweenTrials,as.factor(Repbetween))
colID=with(Rep_betweenTrials,as.factor(ColID))
m6=lmer(log(AverOneTool)~rep+(1|colID),data=Rep_betweenTrials)
summary(m6)

## Linear mixed model fit by REML. t-tests use Satterthwaite's method [
## lmerModLmerTest]
## Formula: log(AverOneTool) ~ rep + (1 | colID)
## Data: Rep_betweenTrials
##
## REML criterion at convergence: 82.8
##
## Scaled residuals:
## Min 1Q Median 3Q Max
## -1.3182 -0.6194 -0.1387 0.2392 2.2226
##
## Random effects:
## Groups Name Variance Std.Dev.
## colID (Intercept) 0.1029 0.3208
## Residual 1.0186 1.0093
## Number of obs: 29, groups: colID, 6
##
## Fixed effects:
## Estimate Std. Error df t value Pr(>|t|)
## (Intercept) 4.603560 0.320969 13.518870 14.343 1.45e-09 ***
## rep2 -0.006188 0.412037 20.316791 -0.015 0.988
## rep3 -0.550688 0.540518 21.579969 -1.019 0.320
## ---
## Signif. codes: 0 '***' 0.001 '**' 0.01 '*' 0.05 '.' 0.1 ' ' 1
##
## Correlation of Fixed Effects:
## (Intr) rep2
## rep2 -0.642
## rep3 -0.495 0.381

Running model diagnostics plot:

plot(m6)


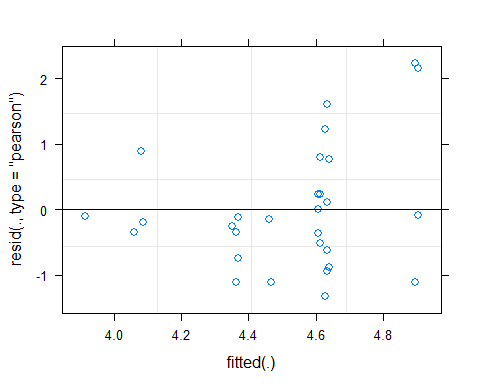


*Model on the effect of ‘interference’ (presence of another worker starting using tools) on a tool*
*user’s performance:*

Work_interfer=read_excel("C:/Users/NITRO/Desktop/RMarkDown/Data_Experiment2.xlsx",sheet=4)

befaf=with(Work_interfer,as.factor(BefAftInterfer))
subID=with(Work_interfer,as.factor(SubcolID))
colID=with(Work_interfer,as.factor(ColID))
m7=lmer(log(TimeOneTool)~befaf+subID+(1|colID),data=Work_interfer)
summary(m7)

## Linear mixed model fit by REML. t-tests use Satterthwaite's method [
## lmerModLmerTest]
## Formula: log(TimeOneTool) ~ befaf + subID + (1 | colID)
## Data: Work_interfer
##
## REML criterion at convergence: 142.7
##
## Scaled residuals:
## Min 1Q Median 3Q Max
## -1.46261 -0.70621 -0.03951 0.64763 2.79801
##
## Random effects:
## Groups Name Variance Std.Dev.
## colID (Intercept) 0.08707 0.2951
## Residual 0.44408 0.6664
## Number of obs: 66, groups: colID, 6
##
## Fixed effects:
## Estimate Std. Error df t value Pr(>|t|)
## (Intercept) 4.08331 0.18702 9.01819 21.834 4.07e-09 ***
## befaf2 0.08177 0.16406 56.90329 0.498 0.620
## subIDSUB 2 -0.08688 0.16661 58.63093 -0.521 0.604
## ---
## Signif. codes: 0 '***' 0.001 '**' 0.01 '*' 0.05 '.' 0.1 ' ' 1
##
## Correlation of Fixed Effects:
## (Intr) befaf2
## befaf2 -0.439
## subIDSUB 2 -0.405 0.000

Running model diagnostics plot:

plot(m7)


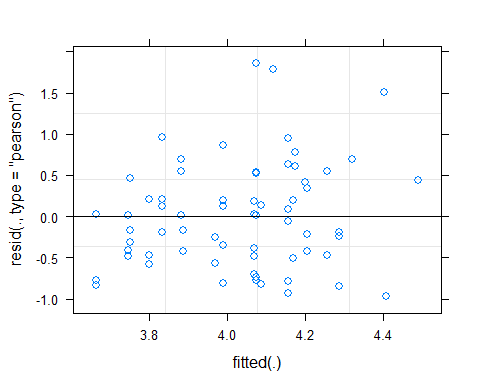


*The effect of the pre-trial on the performance of ants in the subsequent trial: do workers that had the possibility to observe tool users in action (sub-colonies 2) perform better than workers that did not (sub-colonies 1)?*

*Model on the latency of the first tool to the bait:*

Pre_Trial=read_excel("C:/Users/NITRO/Desktop/RMarkDown/Data_Experiment2.xlsx",sheet=5)

colID=with(Pre_Trial,as.factor(ColID))
subID=with(Pre_Trial,as.factor(SubcolID))
m8=lmer(log(FirstTool)~subID+(1|colID),data=Pre_Trial)
summary(m8)

## Linear mixed model fit by REML. t-tests use Satterthwaite's method [
## lmerModLmerTest]
## Formula: log(FirstTool) ~ subID + (1 | colID)
## Data: Pre_Trial
##
## REML criterion at convergence: 36.3
##
## Scaled residuals:
## Min 1Q Median 3Q Max
## -1.40587 -0.55646 0.08544 0.62409 1.06830
##
## Random effects:
## Groups Name Variance Std.Dev.
## colID (Intercept) 0.748 0.8649
## Residual 0.960 0.9798
## Number of obs: 12, groups: colID, 6
##
## Fixed effects:
## Estimate Std. Error df t value Pr(>|t|)
## (Intercept) 6.5219 0.5335 8.3908 12.224 1.23e-06 ***
## subIDsub2 -0.2811 0.5657 5.0000 -0.497 0.64
## ---
## Signif. codes: 0 '***' 0.001 '**' 0.01 '*' 0.05 '.' 0.1 ' ' 1
##
## Correlation of Fixed Effects:
## (Intr)
## subIDsub2 -0.530

Running model diagnostics plot:

plot(m8)


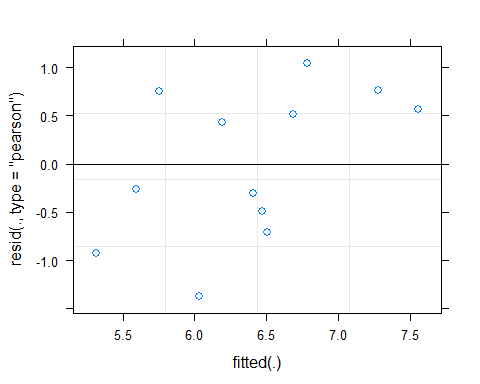


*Model on the total transport time:*

m9=lmer(log(Tottime)~subID+(1|colID),data=Pre_Trial)
summary(m9)

## Linear mixed model fit by REML. t-tests use Satterthwaite's method [
## lmerModLmerTest]
## Formula: log(Tottime) ~ subID + (1 | colID)
## Data: Pre_Trial
##
## REML criterion at convergence: 23
##
## Scaled residuals:
## Min 1Q Median 3Q Max
## -2.2437 -0.2204 0.1865 0.4647 1.4010
##
## Random effects:
## Groups Name Variance Std.Dev.
## colID (Intercept) 0.03734 0.1932
## Residual 0.37298 0.6107
## Number of obs: 12, groups: colID, 6
##
## Fixed effects:
## Estimate Std. Error df t value Pr(>|t|)
## (Intercept) 6.2966 0.2615 9.9180 24.078 3.96e-10 ***
## subIDsub2 -0.3086 0.3526 4.9983 -0.875 0.422
## ---
## Signif. codes: 0 '***' 0.001 '**' 0.01 '*' 0.05 '.' 0.1 ' ' 1
##
## Correlation of Fixed Effects:
## (Intr)
## subIDsub2 -0.674

Running model diagnostics plot:

plot(m9)


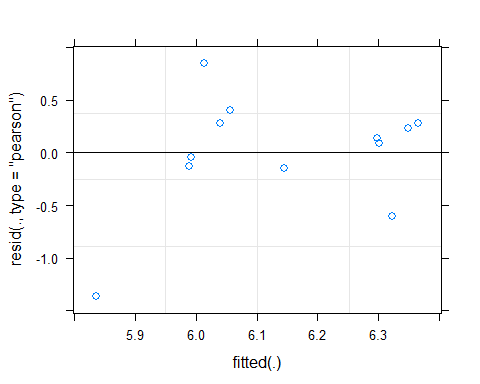


*Model on the number of workers involved in tool use:*

m10=lmer(log(Nr.Work)~subID+(1|colID),data=Pre_Trial)
summary(m10)

## Linear mixed model fit by REML. t-tests use Satterthwaite's method [
## lmerModLmerTest]
## Formula: log(Nr.Work) ~ subID + (1 | colID)
## Data: Pre_Trial
##
## REML criterion at convergence: 19.2
##
## Scaled residuals:
## Min 1Q Median 3Q Max
## -1.9054 -0.4805 0.3272 0.5922 1.0690
##
## Random effects:
## Groups Name Variance Std.Dev.
## colID (Intercept) 0.07379 0.2716
## Residual 0.21622 0.4650
## Number of obs: 12, groups: colID, 6
##
## Fixed effects:
## Estimate Std. Error df t value Pr(>|t|)
## (Intercept) 1.1817 0.2199 9.3920 5.375 0.000387 ***
## subIDsub2 -0.1223 0.2685 5.0000 -0.456 0.667743
## ---
## Signif. codes: 0 '***' 0.001 '**' 0.01 '*' 0.05 '.' 0.1 ' ' 1
##
## Correlation of Fixed Effects:
## (Intr)
## subIDsub2 -0.611

Running model diagnostics plot:

plot(m10)


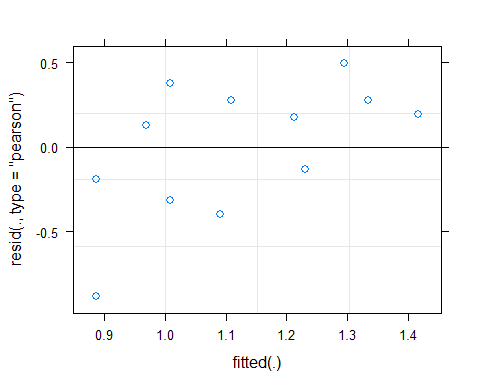


**Experiment 3 - Does worker personality predict tool use?**

*Repeatability across the two sessions of the open-field and the reaction to prey test*

*Model on the time spent walking in the periphery:*

library(lmerTest)
library(rptR)
Exp3_Rep=read_excel("C:/Users/NITRO/Desktop/RMarkDown/Data_Experiment3.xlsx",sheet=1)
rep1=rpt(scale(WalkPeriph)~(1|WorkID)+(1|ColID),adjusted = FALSE,grname = c("WorkID"),data=Exp3_Rep,datatype = "Gaussian",nboot=1000,npermut=0)

## Bootstrap Progress:

## boundary (singular) fit: see ?isSingular

summary(rep1)

##
## Repeatability estimation using the lmm method
##
## Call = rpt(formula = scale(WalkPeriph) ~ (1 | WorkID) + (1 | ColID), grname = c("WorkID"), data = Exp3_Rep, datatype = "Gaussian", nboot = 1000, npermut = 0, adjusted = FALSE)
##
## Data: 308 observations
## ----------------------------------------
##
## WorkID (154 groups)
##
## Repeatability estimation overview:
## R SE 2.5% 97.5% P_permut LRT_P
## 0.273 0.0715 0.13 0.408 NA 0
##
## Bootstrapping and Permutation test:
## N Mean Median 2.5% 97.5%
## boot 1000 0.274 0.276 0.13 0.408
## permut 1 NA NA NA NA
##
## Likelihood ratio test:
## logLik full model = -427.9593
## logLik red. model = -434.4809
## D = 13, df = 1, P = 0.000152
##
## ----------------------------------------

*Model on the total time spent in the central area of the open field:*

rep2=rpt(scale(TotCenter)~(1|WorkID)+(1|ColID),adjusted = FALSE,grname = c("WorkID"),data=Exp3_Rep,datatype = "Gaussian",nboot=1000,npermut=0)

## Bootstrap Progress:

## boundary (singular) fit: see ?isSingular

summary(rep2)

##
## Repeatability estimation using the lmm method
##
## Call = rpt(formula = scale(TotCenter) ~ (1 | WorkID) + (1 | ColID), grname = c("WorkID"), data = Exp3_Rep, datatype = "Gaussian", nboot = 1000, npermut = 0, adjusted = FALSE)
##
## Data: 308 observations
## ----------------------------------------
##
## WorkID (154 groups)
##
## Repeatability estimation overview:
## R SE 2.5% 97.5% P_permut LRT_P
## 0.431 0.0677 0.3 0.555 NA 0
##
## Bootstrapping and Permutation test:
## N Mean Median 2.5% 97.5%
## boot 1000 0.427 0.424 0.3 0.555
## permut 1 NA NA NA NA
##
## Likelihood ratio test:
## logLik full model = -413.7179
## logLik red. model = -432.0826
## D = 36.7, df = 1, P = 6.79e-10
##
## ----------------------------------------

*Model on the time spent in contact with the prey:*

rep3=rpt(ContactPrey~(1|WorkID)+(1|ColID),adjusted = FALSE,grname = c("WorkID"),data=Exp3_Rep,datatype = "Gaussian",nboot=1000,npermut=0)

## Bootstrap Progress:

## boundary (singular) fit: see ?isSingular

summary(rep3)

##
## Repeatability estimation using the lmm method
##
## Call = rpt(formula = ContactPrey ~ (1 | WorkID) + (1 | ColID), grname = c("WorkID"), data = Exp3_Rep, datatype = "Gaussian", nboot = 1000, npermut = 0, adjusted = FALSE)
##
## Data: 308 observations
## ----------------------------------------
##
## WorkID (154 groups)
##
## Repeatability estimation overview:
## R SE 2.5% 97.5% P_permut LRT_P
## 0.614 0.0555 0.491 0.707 NA 0
##
## Bootstrapping and Permutation test:
## N Mean Median 2.5% 97.5%
## boot 1000 0.606 0.609 0.491 0.707
## permut 1 NA NA NA NA
##
## Likelihood ratio test:
## logLik full model = -1537.642
## logLik red. model = -1576.5
## D = 77.7, df = 1, P = 5.95e-19
##
## ----------------------------------------

*Correlation between the time spent walking in the periphery and the time spent in contact with the prey (average of the two sessions for each variable):*

Exp3_RepAver=read_excel("C:/Users/NITRO/Desktop/RMarkDown/Data_Experiment3.xlsx",sheet=2)

with(Exp3_RepAver,cor.test(WalkPeripher,ContactPrey,method="spearman"))

## Warning in cor.test.default(WalkPeripher, ContactPrey, method = "spearman"):
## Cannot compute exact p-value with ties

##
## Spearman's rank correlation rho
##
## data: WalkPeripher and ContactPrey
## S = 789655, p-value = 0.0001807
## alternative hypothesis: true rho is not equal to 0
## sample estimates:
## rho
## -0.2973133

*Correlation between the total time spent in the central area and the time spent in contact with*
*the prey (average of the two sessions for each variable):*

with(Exp3_RepAver,cor.test(TotTimeCenter,ContactPrey,method="spearman"))

## Warning in cor.test.default(TotTimeCenter, ContactPrey, method = "spearman"):
## Cannot compute exact p-value with ties

##
## Spearman's rank correlation rho
##
## data: TotTimeCenter and ContactPrey
## S = 406582, p-value = 2.59e-05
## alternative hypothesis: true rho is not equal to 0
## sample estimates:
## rho
## 0.3320328

*Principal Component Analysis based on the average of the two sessions for each variable:* a) total time spent in the central area of the open field, b) time spent walking in the periphery, c) time spent in contact with the prey

library(FactoMineR)
res.pca=PCA(Exp3_RepAver[,c(3,10:12)],scale.unit=TRUE,ncp=4,quali.sup=c(1),graph=T)


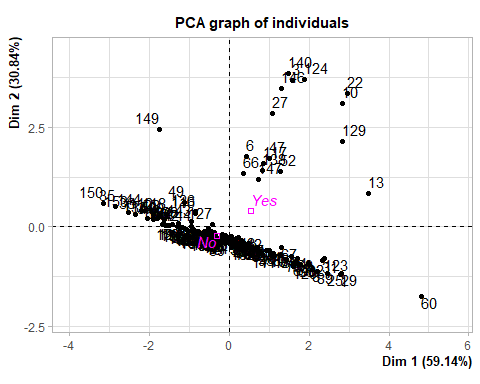


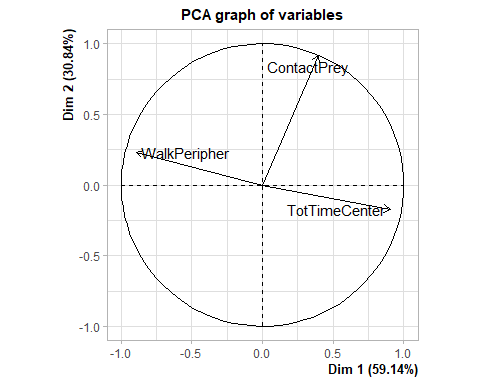


summary(res.pca)

##
## Call:
## PCA(X = Exp3_RepAver[, c(3, 10:12)], scale.unit = TRUE, ncp = 4,
## quali.sup = c(1), graph = T)
##
##
## Eigenvalues
## Dim.1 Dim.2 Dim.3
## Variance 1.774 0.925 0.301
## % of var. 59.145 30.838 10.017
## Cumulative % of var. 59.145 89.983 100.000
##
## Individuals (the 10 first)
## Dist Dim.1 ctr cos2 Dim.2 ctr cos2 Dim.3
## 1 | 3.062 | 2.782 2.833 0.826 | -1.191 0.995 0.151 | 0.465
## 2 | 1.781 | 0.583 0.124 0.107 | -0.537 0.203 0.091 | -1.595
## 3 | 4.048 | 1.593 0.929 0.155 | 3.681 9.512 0.827 | -0.548
## 4 | 0.775 | 0.574 0.121 0.548 | -0.520 0.190 0.450 | -0.031
## 5 | 0.402 | 0.014 0.000 0.001 | -0.401 0.113 0.993 | -0.031
## 6 | 1.846 | 0.415 0.063 0.051 | 1.777 2.215 0.926 | -0.284
## 7 | 0.351 | 0.052 0.001 0.022 | -0.343 0.083 0.955 | -0.053
## 8 | 2.380 | 2.089 1.598 0.771 | -1.069 0.802 0.202 | -0.394
## 9 | 0.427 | 0.172 0.011 0.162 | -0.281 0.056 0.434 | 0.272
## 10 | 4.211 | 2.834 2.939 0.453 | 3.086 6.683 0.537 | -0.425
## ctr cos2
## 1 0.468 0.023 |
## 2 5.499 0.802 |
## 3 0.648 0.018 |
## 4 0.002 0.002 |
## 5 0.002 0.006 |
## 6 0.174 0.024 |
## 7 0.006 0.023 |
## 8 0.335 0.027 |
## 9 0.159 0.404 |
## 10 0.391 0.010 |
##
## Variables
## Dim.1 ctr cos2 Dim.2 ctr cos2 Dim.3 ctr
## TotTimeCenter | 0.904 46.076 0.818 | -0.174 3.267 0.030 | 0.390 50.658
## WalkPeripher | -0.893 44.970 0.798 | 0.233 5.875 0.054 | 0.384 49.155
## ContactPrey | 0.399 8.954 0.159 | 0.917 90.858 0.841 | -0.024 0.188
## cos2
## TotTimeCenter 0.152 |
## WalkPeripher 0.148 |
## ContactPrey 0.001 |
##
## Supplementary categories
## Dist Dim.1 cos2 v.test Dim.2 cos2 v.test Dim.3
## No | 0.387 | -0.312 0.650 -3.838 | -0.224 0.336 -3.819 | 0.046
## Yes | 0.678 | 0.547 0.650 3.838 | 0.393 0.336 3.819 | -0.080
## cos2 v.test
## No 0.014 1.366 |
## Yes 0.014 -1.366 |

plotellipses(res.pca,graph.type="ggplot")


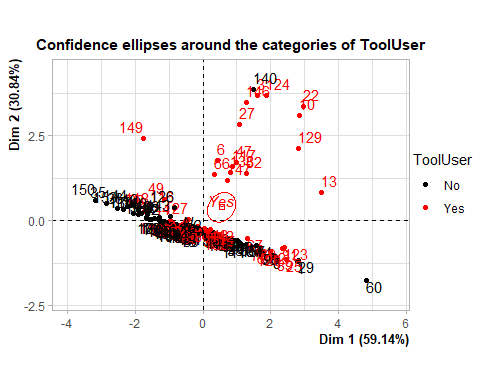


*Model on the link between personality score and tool use behaviour:*

tooluser=with(Exp3_RepAver,as.factor(ToolUser))
colID=with(Exp3_RepAver,as.factor(ColID))
m11=glmer(tooluser~PersScore+(1|colID),family=binomial,data=Exp3_RepAver)

## boundary (singular) fit: see ?isSingular

summary(m11)

## Generalized linear mixed model fit by maximum likelihood (Laplace
## Approximation) [glmerMod]
## Family: binomial ( logit )
## Formula: tooluser ~ PersScore + (1 | colID)
## Data: Exp3_RepAver
##
## AIC BIC logLik deviance df.resid
## 189.3 198.4 -91.6 183.3 151
##
## Scaled residuals:
## Min 1Q Median 3Q Max
## -2.7203 -0.7097 -0.5091 0.9735 2.4816
##
## Random effects:
## Groups Name Variance Std.Dev.
## colID (Intercept) 0 0
## Number of obs: 154, groups: colID, 8
##
## Fixed effects:
## Estimate Std. Error z value Pr(>|z|)
## (Intercept) -0.6226 0.1803 -3.453 0.000555 ***
## PersScore 0.6174 0.1555 3.972 7.14e-05 ***
## ---
## Signif. codes: 0 '***' 0.001 '**' 0.01 '*' 0.05 '.' 0.1 ' ' 1
##
## Correlation of Fixed Effects:
## (Intr)
## PersScore -0.147
## convergence code: 0
## boundary (singular) fit: see ?isSingular

Running model diagnostics plot:

plot(m11)


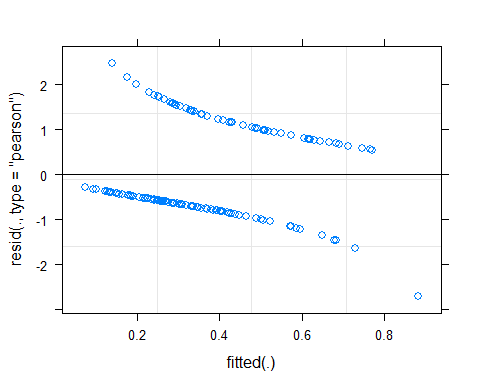


Checking model for overdispersion:

modeloverdisp<- function(model) {
 rdf <- df.residual(model)
 rp <- residuals(model,type="pearson")
 Pearson.chisq <- sum(rp^2)
 prat <- Pearson.chisq/rdf
 pval <- pchisq(Pearson.chisq, df=rdf, lower.tail=FALSE)
 c(chisq=Pearson.chisq,ratio=prat,rdf=rdf,p=pval)
}
modeloverdisp(m11)

## chisq ratio rdf p
## 153.9649908 1.0196357 151.0000000 0.4176831

Our model is not overdispersed.

Calculating the Cohen.d to assess the effect size for the different factor levels:

tooluseYes=subset(Exp3_RepAver,ToolUser=="Yes")
tooluseNo=subset(Exp3_RepAver,ToolUser=="No")

cohen.d(tooluseYes$PersScore,tooluseNo$PersScore,conf.level=0.95)

##
## Cohen's d
##
## d estimate: 0.7492026 (medium)
## 95 percent confidence interval:
## lower upper
## 0.4076667 1.0907386

*The correlations between the latency to find the prey and the time spent in contact with the prey measured during the two sessions of the reaction to prey test. Analysis are performed generally (using all the ants) and also separately for the tool user and non-tool user workers:*

Lat_contact_prey=read_excel("C:/Users/NITRO/Desktop/RMarkDown/Data_Experiment3.xlsx",sheet=3)

with(Lat_contact_prey,cor.test(Lat1,ContactPrey1,method="spearman"))

## Warning in cor.test.default(Lat1, ContactPrey1, method = "spearman"): Cannot
## compute exact p-value with ties

##
## Spearman's rank correlation rho
##
## data: Lat1 and ContactPrey1
## S = 446765, p-value = 0.0008543
## alternative hypothesis: true rho is not equal to 0
## sample estimates:
## rho
## 0.2660166

with(Lat_contact_prey,cor.test(Lat2,ContactPrey2,method="spearman"))

## Warning in cor.test.default(Lat2, ContactPrey2, method = "spearman"): Cannot
## compute exact p-value with ties

##
## Spearman's rank correlation rho
##
## data: Lat2 and ContactPrey2
## S = 355305, p-value = 7.896e-08
## alternative hypothesis: true rho is not equal to 0
## sample estimates:
## rho
## 0.4162747

Lat_contact_prey_ToolUser=subset(Lat_contact_prey,ToolUser=="Yes")
with(Lat_contact_prey_ToolUser,cor.test(Lat1,ContactPrey1,method="spearman"))

## Warning in cor.test.default(Lat1, ContactPrey1, method = "spearman"): Cannot
## compute exact p-value with ties

##
## Spearman's rank correlation rho
##
## data: Lat1 and ContactPrey1
## S = 24773, p-value = 0.2591
## alternative hypothesis: true rho is not equal to 0
## sample estimates:
## rho
## 0.1533638

with(Lat_contact_prey_ToolUser,cor.test(Lat2,ContactPrey2,method="spearman"))

## Warning in cor.test.default(Lat2, ContactPrey2, method = "spearman"): Cannot
## compute exact p-value with ties

##
## Spearman's rank correlation rho
##
## data: Lat2 and ContactPrey2
## S = 23370, p-value = 0.1368
## alternative hypothesis: true rho is not equal to 0
## sample estimates:
## rho
## 0.2013119

Lat_contact_prey_NonToolUser=subset(Lat_contact_prey,ToolUser=="No")
with(Lat_contact_prey_NonToolUser,cor.test(Lat1,ContactPrey1,method="spearman"))

## Warning in cor.test.default(Lat1, ContactPrey1, method = "spearman"): Cannot
## compute exact p-value with ties

##
## Spearman's rank correlation rho
##
## data: Lat1 and ContactPrey1
## S = 99851, p-value = 0.0002352
## alternative hypothesis: true rho is not equal to 0
## sample estimates:
## rho
## 0.3633928

with(Lat_contact_prey_NonToolUser,cor.test(Lat2,ContactPrey2,method="spearman"))

## Warning in cor.test.default(Lat2, ContactPrey2, method = "spearman"): Cannot
## compute exact p-value with ties

##
## Spearman's rank correlation rho
##
## data: Lat2 and ContactPrey2
## S = 67523, p-value = 9.388e-10
## alternative hypothesis: true rho is not equal to 0
## sample estimates:
## rho
## 0.5695014

**The effect of the removal of tool users on the characteristics of tool transport in the subsequent trials: are the new tool users less efficient than the previous ones (which were**
**removed)?**

*Model on the latency to drop the first tool into the bait:*

Exp3_Trial=read_excel("C:/Users/NITRO/Desktop/RMarkDown/Data_Experiment3.xlsx",sheet=4)

trial=with(Exp3_Trial,as.factor(NrTrial))
colID=with(Exp3_Trial,as.factor(ColID))
m12=lmer(FirsttoBait~trial+(1|colID),data=Exp3_Trial)

## boundary (singular) fit: see ?isSingular

summary(m12)

## Linear mixed model fit by REML. t-tests use Satterthwaite's method [
## lmerModLmerTest]
## Formula: FirsttoBait ~ trial + (1 | colID)
## Data: Exp3_Trial
##
## REML criterion at convergence: 222.1
##
## Scaled residuals:
## Min 1Q Median 3Q Max
## -1.3659 -0.7594 -0.3381 0.6515 2.3920
##
## Random effects:
## Groups Name Variance Std.Dev.
## colID (Intercept) 0.0 0.00
## Residual 441.9 21.02
## Number of obs: 28, groups: colID, 8
##
## Fixed effects:
## Estimate Std. Error df t value Pr(>|t|)
## (Intercept) 41.500 7.432 24.000 5.584 9.56e-06 ***
## trial2 1.214 10.880 24.000 0.112 0.912
## trial3 10.214 10.880 24.000 0.939 0.357
## trial4 -8.000 11.353 24.000 -0.705 0.488
## ---
## Signif. codes: 0 '***' 0.001 '**' 0.01 '*' 0.05 '.' 0.1 ' ' 1
##
## Correlation of Fixed Effects:
## (Intr) trial2 trial3
## trial2 -0.683
## trial3 -0.683 0.467
## trial4 -0.655 0.447 0.447
## convergence code: 0
## boundary (singular) fit: see ?isSingular

Running model diagnostics plot:

plot(m12)


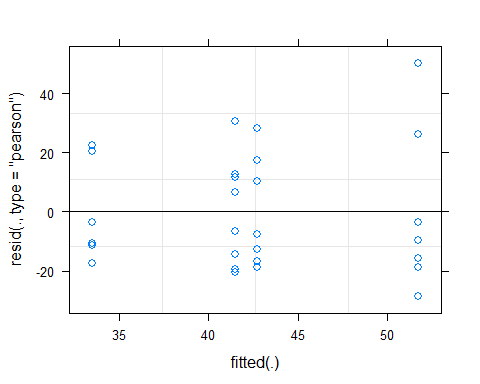


*Model on the total transport time:*

m13=lmer(TotTime~trial+(1|colID),data=Exp3_Trial)

## boundary (singular) fit: see ?isSingular

summary(m13)

## Linear mixed model fit by REML. t-tests use Satterthwaite's method [
## lmerModLmerTest]
## Formula: TotTime ~ trial + (1 | colID)
## Data: Exp3_Trial
##
## REML criterion at convergence: 331.8
##
## Scaled residuals:
## Min 1Q Median 3Q Max
## -1.1510 -0.7867 -0.3462 0.6586 2.3896
##
## Random effects:
## Groups Name Variance Std.Dev.
## colID (Intercept) 0 0
## Residual 42859 207
## Number of obs: 28, groups: colID, 8
##
## Fixed effects:
## Estimate Std. Error df t value Pr(>|t|)
## (Intercept) 414.62 73.19 24.00 5.665 7.81e-06 ***
## trial2 12.09 107.15 24.00 0.113 0.911
## trial3 109.66 107.15 24.00 1.023 0.316
## trial4 -78.62 111.81 24.00 -0.703 0.489
## ---
## Signif. codes: 0 '***' 0.001 '**' 0.01 '*' 0.05 '.' 0.1 ' ' 1
##
## Correlation of Fixed Effects:
## (Intr) trial2 trial3
## trial2 -0.683
## trial3 -0.683 0.467
## trial4 -0.655 0.447 0.447
## convergence code: 0
## boundary (singular) fit: see ?isSingular

Running model diagnostics plot:

plot(m13)


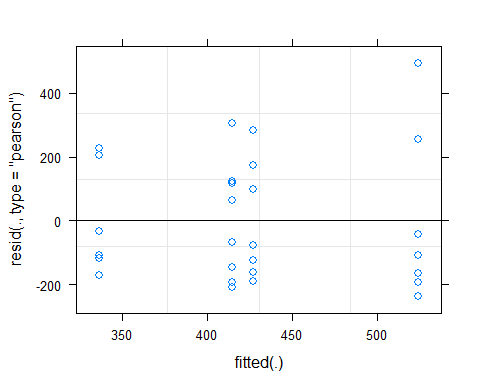


*Model on the number of workers involved in tool use:*

m14=glmer(Nr.Work~trial+(1|colID),data=Exp3_Trial,family="poisson")

## boundary (singular) fit: see ?isSingular

summary(m14)

## Generalized linear mixed model fit by maximum likelihood (Laplace
## Approximation) [glmerMod]
## Family: poisson ( log )
## Formula: Nr.Work ~ trial + (1 | colID)
## Data: Exp3_Trial
##
## AIC BIC logLik deviance df.resid
## 74.4 81.0 -32.2 64.4 23
##
## Scaled residuals:
## Min 1Q Median 3Q Max
## -0.3198 -0.2887 -0.2520 0.0000 1.3858
##
## Random effects:
## Groups Name Variance Std.Dev.
## colID (Intercept) 0 0
## Number of obs: 28, groups: colID, 8
##
## Fixed effects:
## Estimate Std. Error z value Pr(>|z|)
## (Intercept) 0.31845 0.30151 1.056 0.291
## trial2 -0.31845 0.48349 -0.659 0.510
## trial3 -0.06714 0.44947 -0.149 0.881
## trial4 -0.03077 0.46466 -0.066 0.947
##
## Correlation of Fixed Effects:
## (Intr) trial2 trial3
## trial2 -0.624
## trial3 -0.671 0.418
## trial4 -0.649 0.405 0.435
## convergence code: 0
## boundary (singular) fit: see ?isSingular

Checking model for overdispersion:

modeloverdisp(m14)

## chisq ratio rdf p
## 4.9292929 0.2143171 23.0000000 0.9999753

Our model is not overdispersed.

Running model diagnostics plot:

plot(m14)


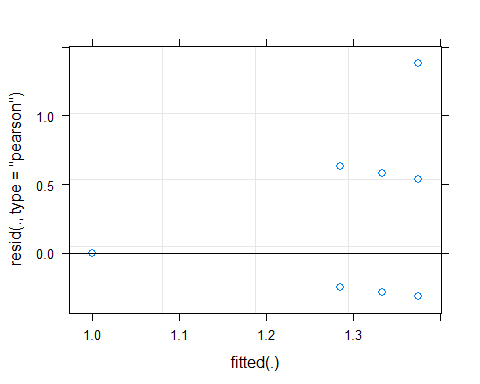

Supplement: Source code 1. [file elife-61298-code1.docx.zip › Rmarkdown_Text.docx]
