# Supplementary material for: A small number of workers with specific personality traits perform tool use in ants
Source: eLife. 2020 Dec 9;9:e61298. doi: 10.7554/eLife.61298 (PMC7725502; doi:10.7554/eLife.61298)
Supplement: Transparent reporting form [file elife-61298-transrepform.docx]

***eLife’s* transparent reporting form**

We encourage authors to provide detailed information *within their submission* to facilitate the interpretation and replication of experiments. Authors can upload supporting documentation to indicate the use of appropriate reporting guidelines for health-related research (see [EQUATOR Network](http://www.equator-network.org/%20)), life science research (see the [BioSharing Information Resource](https://biosharing.org/)), or the [ARRIVE guidelines](http://www.plosbiology.org/article/info:doi/10.1371/journal.pbio.1000412) for reporting work involving animal research. Where applicable, authors should refer to any relevant reporting standards documents in this form.

If you have any questions, please consult our Journal Policies and/or contact us: [editorial@elifesciences.org](mailto:editorial@elifesciences.org).

**Sample-size estimation**

- You should state whether an appropriate sample size was computed when the study was being designed
- You should state the statistical method of sample size computation and any required assumptions
- If no explicit power analysis was used, you should describe how you decided what sample (replicate) size (number) to use

Please outline where this information can be found within the submission (e.g., sections or figure legends), or explain why this information doesn’t apply to your submission:

It was impossible to pre-determine how many workers would participate in tool use in the different experiments.

The information about sample size is stated in the Method section and reported together with the statistical results in the Results section, for example:

(2.86 ± 0.12 workers, mean ± SE; LMM t = 2.41, N = 22, p = 0.02).

In Experiment 1 we used 3 ant colonies. in Experiment 2 we used 6 source colonies and we created two sub-colonies from each of them. Each sub-colony contained 40 workers. In Experiment 3 we used 8 source colonies, with a test-sub-colony from each on them, which contained 20 workers. This information is in the Method section.

**Replicates**

- You should report how often each experiment was performed
- You should include a definition of biological versus technical replication
- The data obtained should be provided and sufficient information should be provided to indicate the number of independent biological and/or technical replicates
- If you encountered any outliers, you should describe how these were handled
- Criteria for exclusion/inclusion of data should be clearly stated
- High-throughput sequence data should be uploaded before submission, with a private link for reviewers provided (these are available from both GEO and ArrayExpress)

Please outline where this information can be found within the submission (e.g., sections or figure legends), or explain why this information doesn’t apply to your submission:

Experiment 1: we performed 5 replicates for each colony, this information is in the Method section and in Table 1.

Experiment 2: the experimental procedure is described in the Method section and in Figure 1.

Experiment 3: the experimental procedure is described in the Method section and in Figure 4.

We did not exclude any data.

In Experiment 3 we had 8 sub-colonies with 20 workers each (160 workers). Workers were tested individually in two personality tests, repeated twice. Between the first and the second replication 6 workers died, therefore the sample size for the repeatability of the personality tests and the link with between tool use behavior and personality is 154 workers.

**Statistical reporting**

- Statistical analysis methods should be described and justified
- Raw data should be presented in figures whenever informative to do so (typically when N per group is less than 10)
- For each experiment, you should identify the statistical tests used, exact values of N, definitions of center, methods of multiple test correction, and dispersion and precision measures (e.g., mean, median, SD, SEM, confidence intervals; and, for the major substantive results, a measure of effect size (e.g., Pearson's r, Cohen's d)
- Report exact p-values wherever possible alongside the summary statistics and 95% confidence intervals. These should be reported for all key questions and not only when the p-value is less than 0.05.

Please outline where this information can be found within the submission (e.g., sections or figure legends), or explain why this information doesn’t apply to your submission:

The statistical analyses are clearly described in the Method section and the codes used for data analysis in R are given as additional file, together with the full model outputs.

Data are reported as mean ± SE or mean and CI_95%_.

(For large datasets, or papers with a very large number of statistical tests, you may upload a single table file with tests, Ns, etc., with reference to sections in the manuscript.)

**Group allocation**

- Indicate how samples were allocated into experimental groups (in the case of clinical studies, please specify allocation to treatment method); if randomization was used, please also state if restricted randomization was applied
- Indicate if masking was used during group allocation, data collection and/or data analysis

Please outline where this information can be found within the submission (e.g., sections or figure legends), or explain why this information doesn’t apply to your submission:

N/A

**Additional data files (“source data”)**

- We encourage you to upload relevant additional data files, such as numerical data that are represented as a graph in a figure, or as a summary table
- Where provided, these should be in the most useful format, and they can be uploaded as “Source data” files linked to a main figure or table
- Include model definition files including the full list of parameters used
- Include code used for data analysis (e.g., R, MatLab)
- Avoid stating that data files are “available upon request”

Please indicate the figures or tables for which source data files have been provided:

We uploaded additional files with the original data used for analysis and to prepare the figures. We uploaded a file with the R codes and model outputs used of data analysis.
